# Complete Genome Sequence of Francisella tularensis subsp. *holarctica* Strain A271_1 (FDC408), Isolated from a Eurasian Beaver (Castor fiber)

**DOI:** 10.1128/MRA.00738-20

**Published:** 2020-11-05

**Authors:** David Sundell, Ingrid Uneklint, Caroline Öhrman, Emelie Salomonsson, Linda Karlsson, Stina Bäckman, Jonas Näslund, Andreas Sjödin, Mats Forsman, Sandra Appelt, Oliver Drechsel, Daniela Jacob, Klaus Heuner, Kerstin Myrtennäs

**Affiliations:** a Division of CBRN Defence and Security, Swedish Defence Research Agency (FOI), Umeå, Sweden; b Centre for Biological Threats and Special Pathogens (ZBS2), Robert Koch Institute (RKI), Berlin, Germany; c Methodology and Research Infrastructure Bioinformatics (MF1), Robert Koch Institute (RKI), Berlin, Germany; d Cellular Interactions of Bacterial Pathogens (ZBS2), Robert Koch Institute (RKI), Berlin, Germany; University of Southern California

## Abstract

Here, we report the complete genome sequence of Francisella tularensis subsp. *holarctica* strain A271_1, isolated from a Eurasian beaver (*Castor fiber*) in 2012 in the Berlin/Brandenburg region, Germany.

## ANNOUNCEMENT

Tularemia is a zoonosis caused by Francisella tularensis (1). Susceptibility to this disease differs between the Eurasian beaver (*Castor fiber*) and the American beaver (*Castor canadensis*), where the latter is more sensitive (2 – 4).

Here, we present the complete genome sequence of F. tularensis subsp. *holarctica* strain A271_1. For further information about the isolation, cultivation, draft genome sequence, and further characterization of this strain, see reference 5. Prior to sequencing, the strain was cultured from the original glycerol stock (−80°C), and DNA was extracted as previously described (5). Human serum (Seraclot, PAN-Biotech, Germany) was spiked with DNA and diluted 1:10 in phosphate-buffered saline (PBS) to an unmeasurable genomic DNA concentration on both a Qubit fluorometer (Thermo Fisher Scientific, USA) and a NanoDrop 2000 spectrophotometer (Thermo Fisher Scientific). DNA was extracted using MagAttract (Qiagen, Germany) and amplified using a REPLI-g midikit (Qiagen) to a concentration of 1,518 ng/μl. The first sequencing library was prepared with the Nextera XT DNA library prep kit v3 (Illumina, Inc., USA) and sequenced on a MiSeq instrument (Illumina, Inc.) using a MiSeq reagent kit v3 (600 cycles). After endonuclease treatment of the amplified DNA with T7 endonuclease I (New England Biolabs, USA), the second sequencing library was prepared using a ligation sequencing kit (SQK-LSK108) with the native barcoding expansion kit (EXP-NBD103) and sequenced using a MinION FLO-MIN107 flow cell with R9.4 chemistry (Oxford Nanopore Technologies, Ltd., UK). No DNA size selection or fragmentation was performed prior to sequencing. The sequencing generated 4,275,968 trimmed paired-end reads (300 bp) and 424,221 long reads (median read length, 1,763 bp; read length *N*_50_, 4,027 bp). Illumina reads were trimmed using Trimmomatic v0.36 (LEADING:3, TRAILING:3, SLIDINGWINDOW:4:15, MINLEN:36), removing the low-quality bases at the ends of reads (6). Nanopore reads were base called using Albacore v2.1.3 (Oxford Nanopore Technologies, Ltd.), and adapters were trimmed with Porechop v0.2.3_seqan2.1.1 (7). One 1,862,390-bp linear sequence was generated with Unicycler v0.4.7, using both short Illumina reads and long Nanopore reads as input (8). The coverage of the 30-kb *Francisella* pathogenicity island (FPI) was twice as high as the average coverage (>100×) of the genome, indicating a duplicated region. This finding was expected since the FPI is duplicated in all known genomes of this subspecies (9). The second copy of the FPI was added, guided by the complete genome of strain FSC200 (GenBank accession no. CP003862.1) (10). By mapping Illumina reads using Bowtie 2 v2.3.4.3 (10) and Nanopore reads longer than 10 kbp using minimap2 v2.15 (11) to the complete genome of A271_1 with two FPI copies (12), we could confirm having both long and short reads spanning all four junctions of the FPIs with over 10× coverage without any introduced single nucleotide polymorphisms (SNPs) (13). Finally, the genome was rotated to start with *dnaA* using Circlator v1.5.5 (14). Annotation was performed using the NCBI Prokaryotic Genome Annotation Pipeline (22 January 2020) (15, 16). We confirmed with CanSNPer2 (17) (https://github.com/FOI-Bioinformatics/CanSNPer2) that the strain is a member of the B.12 subclade B.75. Default parameters were used for all software unless otherwise specified.

The 1,893,679-bp circular chromosome of A271_1 has a GC content of 32.16% with 1,591 protein-coding sequences, 280 pseudogenes, 10 rRNAs, 38 tRNAs, and 4 noncoding RNAs. The new assembly contained 11 single base alternates and one single base insertion, compared to the draft genome (accession no. GCA_002102455.1). The changes were identified by dnadiff v1.3 and were carefully verified with the read data using IGV v2.3. All differences were located near insertion elements (IS) and within 218 bp from the contig ends, regions known to be problematic for assemblies based solely on Illumina sequences.

### Data availability.

The complete genome sequence of strain A271_1 (BioProject accession no. PRJNA602379) has been deposited in GenBank under the accession no. CP048229.1 and the SRA accession no. SRR11853651 (Oxford Nanopore) and SRR11853652 (Illumina). The version described in this paper is the second version. The first version of the A271_1 (FDC408) genome sequence (BioProject accession no. PRJNA285142) was a draft genome sequence and has the GenBank and SRA accession no. GCA_002102455.1 and SRR5051916, respectively.
